# Resilience Among Dementia Family Caregivers in a Changing Social World

**DOI:** 10.1093/geroni/igab046.032

**Published:** 2021-12-17

**Authors:** Brandy McCann, Karen Roberto, Tina Savla, Rosemary Blieszner, Emily Hoyt

**Affiliations:** Virginia Tech, Blacksburg, Virginia, United States

## Abstract

Dementia caregivers must manage the social worlds of their loved ones as well as their own. In a mixed methods study, we interviewed 50 family caregivers prior to the pandemic, twice during early phases of the pandemic, and again during the vaccine roll-out phase. Findings revealed how implementation of stay-at-home orders altered reliance on informal support as well as social ties and interactions. Using content analysis, we identified three ways in which caregivers’ managed changes in their social world: rethinking family visits (fewer people, higher quality); reinventing public spaces (church services, exercise venues); and reconsidering self-care (setting boundaries, solace in nature). Caregivers showed varying degrees of resilience in the ways they managed adverse social situations and cared for themselves. Findings reinforce the need for inclusive programs and services to help caregivers learn to maintain supportive social connections that reinforce their care decisions and routines, particularly during times of duress.

